# The Effect of Solvent Hydrophilicity on the Enzymatic Ring-Opening Polymerization of L-Lactide by *Candida rugosa* Lipase

**DOI:** 10.3390/polym14183856

**Published:** 2022-09-15

**Authors:** Catia Angli Curie, Muhammad Arif Darmawan, Dianursanti Dianursanti, Wiratni Budhijanto, Misri Gozan

**Affiliations:** 1 Chemical Engineering Department, Faculty of Engineering, Universitas Indonesia, Depok 16424, Indonesia; 2 Department of Chemical Engineering, Universitas Pertamina, Jakarta 12220, Indonesia; 3 Research Center for Process and Manufacturing Industry Technology, Research Organization for Energy and Manufacture, National Research and Innovation Agency, South Tangerang 15314, Indonesia; 4 Bioprocess Engineering Program, Department of Chemical Engineering, Faculty of Engineering, Universitas Indonesia, Depok 16424, Indonesia; 5 Chemical Engineering Department, Faculty of Engineering, Universitas Gadjah Mada, Yogyakarta 55281, Indonesia

**Keywords:** enzymatic ring-opening polymerization (eROP), polylactic acid, L-lactide, *Candida rugosa* lipase, hydrophilicity, hydrophobicity, organic solvents, ionic liquid, [BMIM][PF_6_], [PF_6_]^−^ hydrolysis

## Abstract

Contradictions have been reported on the effect of organic solvents, especially toluene, on enzymatic ring-opening polymerization (eROP) of L-lactide. Studies have shown that log P, a common measure of hydrophilicity, affects enzyme activity. This study examines the effect of solvents with various log P values on the eROP of L-lactide, performed using *Candida rugosa* lipase (CRL). N,N-dimethylacetamide (DMA), 1,2-dimethoxybenzene, 1,4-dimethoxybenzene, diphenyl ether, and dodecane were used as the organic solvents. The eROP in ionic liquid 1-butyl-3-methylimidazolium hexafluorophosphate ([BMIM][PF_6_]) was also conducted to compare its performance with the organic solvents. The results show that [BMIM][PF_6_]-mediated eROP gave better conversion and molecular weight than the organic solvent-mediated eROP. In this study, the effects of solvents hydrophilicity are discussed, including the possibility of hexafluorophosphate ion ([PF_6_]^−^) hydrolysis to occur.

## 1. Introduction

Poly-L-lactic acid (PLLA) has been widely known for its biocompatibility and biodegradability. The degradation products of PLLA are non-toxic and can be released from the body through the Kreb cycle, which renders PLLA suitable for biomedical applications [1,2,3,4]. The properties of PLA can be tailored, for example, by choosing the different isomers as the monomer, setting the achieved molecular weight, blending, co-polymerization, and functionalization [1,5]. Enzymatic ring-opening polymerization (eROP) of lactides with various lipases, in bulk and a solvent, has also emerged as an option for a greener synthesis mode [6,7,8,9,10,11,12,13,14,15,16,17].

Several things remained unclear on the effect of solvent hydrophilicity on the eROP of L-lactide. A classical study by Laane et al. (1987) shows that, generally, enzymatic activity in organic solvents follows an S-shaped behavior linked to the log P values of the surrounding solvent. Log P, the partition coefficient’s logarithm, indicates a compound’s preference to dissolve in a hydrophobic or hydrophilic environment. Generally, the partition coefficient is determined by comparing the concentration of the measured compound in octanol (as the hydrophobic media) with its concentration in water. A stronger preference for water would give a lower log P value, which indicates the compound’s hydrophilicity. Hydrophobic compounds are those that have higher concentrations in octanol than in water, giving a high log P value. Enzymes in hydrophilic solvents (log P < 2.0) usually exhibit low activity since the solvents induce enzyme denaturation as they draw the essential water away from the enzymes. Hydrophobic solvents (log P > 4.0) preserve enzymatic activity because they keep the enzymes hydrated by leaving their essential water intact. In contrast, those whose log P is in between gave unpredictable results [18]. Similar trends were observed on the eROP of ε-caprolactone (ε-CL) by lipase PS and Novozym-435. Among the solvents studied, toluene was preferred because it could give a high number average molecular weight (Mn) and ε-CL conversion [19,20]. On the contrary, eROP of L-lactide in toluene by the same type of lipases yielded low molecular weight PLA (Mn ≤ 1000 g/mol) and even no polymerization [9,14,21]. A more recent study showed that one of the best solvents for the eROP of L-lactide was DMA which facilitated the formation of high-weight average molecular weight PLA (Mw >10,000 g/mol), with high monomer conversion as well as acceptable yield and dispersity. According to them, DMA can maintain Novozym-435 activity to a certain degree because the enzymes could still be reused after an extreme condition [15]. Nevertheless, DMA is a hydrophilic solvent (log P −0.77 [22]). As described, earlier studies suggested that hydrophilic solvents normally lead to low enzyme activity and low polymerization [18,19,20]. Because of those contradicting reports, this study is intended to evaluate the effect of organic solvent hydrophilicity, measured as log P, on the eROP of L-lactide catalyzed by *Candida rugosa* lipase (CRL).

Ionic liquids have also become emerging solvents for use in eROP due to their excellent properties, which include non-volatility; the ability to dissolve broad kinds of compounds, including those that are insoluble in many organic solvents; tailorable properties; and thermally stable [23,24,25]. An eROP of L-lactide study that used several ionic liquids, including [BMIM][PF_6_] and 1-butyl-3-methylimidazolium tetrafluoroborate ([BMIM][BF_4_]), showed that the highest Mw of PLA (17,000 g/mol) with acceptable yield (20%) was achieved using [BMIM][PF_6_] [15]. Nonetheless, another study observed that significantly lower PLA molecular weight (3600 g/mol) was obtained from [BMIM][PF_6_]-mediated eROP than that mediated by [BMIM][BF_4_] (54,600 g/mol) [8]. Those studies used *Candida antarctica* lipase B (CALB) as the biocatalyst in its free or immobilized forms. On the other hand, studies indicated that with regard to CRL, [BMIM][PF_6_] seems to have good compatibility compared to more hydrophilic ionic liquids [26]. CRL also exhibited better activity and thermal stability in [BMIM][PF_6_] than in n-hexane, a commonly well-performing organic solvent in typical enzymatic reactions [26,27]. Therefore, to find a better solvent for the CRL-catalyzed eROP of L-lactide, it is reasonable to study the use of [BMIM][PF_6_] and compare the result with the studied organic solvent-mediated eROP.

## 2. Materials and Methods

### 2.1. Materials

L-lactide, *Candida rugosa* lipases (CRL) (catalogue #62316), N,N-dimethylacetamide (DMA), 1,2-dimethoxybenzene, 1,4-dimethoxybenzene, diphenyl ether, and 1-butyl-3-methylimidazolium hexafluorophosphate ([BMIM][PF_6_]) were purchased from Sigma-Aldrich (St. Louis, MO, USA). Dodecane, chloroform, and methanol were obtained from Merck KGaA (Darmstadt, Germany). The solvents used were of ≥99% purity, while chloroform and methanol were of EMSURE grade.

### 2.2. Enzymatic Ring-Opening Polymerization (eROP) of L-lactide

**Figure 1:**
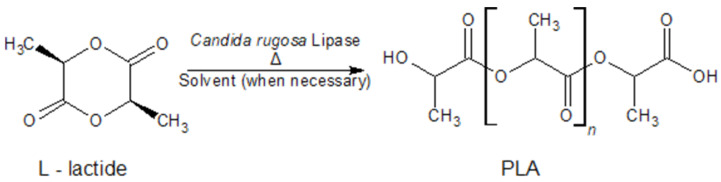
Reaction scheme of the eROP of L-lactide.

For the polymerization, 2.5 g of L-lactide was put in a reaction tube with a pre-determined amount of *Candida rugosa* lipase (CRL). The solvent effect was studied by adding 1.25 mL of it, unless specified otherwise, into the reaction vessel, while the bulk eROP was performed without adding the solvent. The reaction mixture was then purged with Argon at room temperature for 10 min to push out air from the reaction system. The reaction mixture was placed in an oil bath at the desired temperature, where the reaction mixture started to melt (in bulk eROP)/homogenized (for solvent-mediated eROP) and allowed to react according to Figure 1 for 72 h.

The reaction was halted by quenching the tube in water at room temperature. Chloroform was added to the reaction mixture to dissolve the PLA formed and the remaining lactide. This mixture was centrifuged to separate the CRL. Some samples were taken from the supernatant for ^1^H-NMR analysis, while most were precipitated in ice-cold methanol to obtain the solid product. The obtained product was centrifuged and dried in a vacuum desiccator at room temperature.

### 2.3. Characterizations

^1^H-NMR and ^13^C-NMR analysis were performed using Nuclear Magnetic Resonance Spectrometer-Bruker Avance Neo 500 MHz (Bruker Corporation, Billerica, MA, USA). The samples were dissolved in deuterated chloroform prior to the analysis. The ^1^H-NMR spectra were used to estimate the monomer conversion according to the method described by Duchiron et al. [14].

Gel permeation chromatography (GPC) was used to determine the molecular weight and polydispersity of the precipitated samples. The GPC was performed using Shimadzu LC-20, with UV-RID detector and LF 804 column (Shimadzu Corporation, Kyoto, Japan). Tetrahydrofuran (THF) was used as the solvent, and polystyrene was used as the standard.

## 3. Results and Discussion

### 3.1. The Ability of Candida rugosa Lipase (CRL) to Catalyze Enzymatic Ring-Opening Polymerization (eROP) of L-lactide

Compared to lipase B from *Candida antarctica* (CALB/Novozym-435) and lipase from *Burkholderia cepacia* (lipase PS/BCL), *Candida rugosa* lipase has been considerably less studied for the eROP of L-lactide. Bulk eROP of commercial L-lactide at 100 °C was performed in this study to assess CRL activity in catalyzing the polymerization. A lower temperature was not possible since L-lactide only started to melt at around 98 °C, which is in line with the reported thermal properties of L-lactide [28].

The eROP here was performed in a closed batch reaction tube, where the lower part of the tube was submerged in an oil bath while the upper part was in contact with ambient air. With this configuration, crystals formed in the upper part of the tube during the eROP process. These crystals seem to be formed from the vapor of the reaction mixture, i.e., lactide melt in the case of bulk eROP, that was cooled as it reached the upper part of the reaction tube. ^1^H-NMR of the crystals from bulk ROP shows that the crystals are indeed lactide. The mass of the crystals formed has been considered when determining monomer conversion.

**Figure 2:**
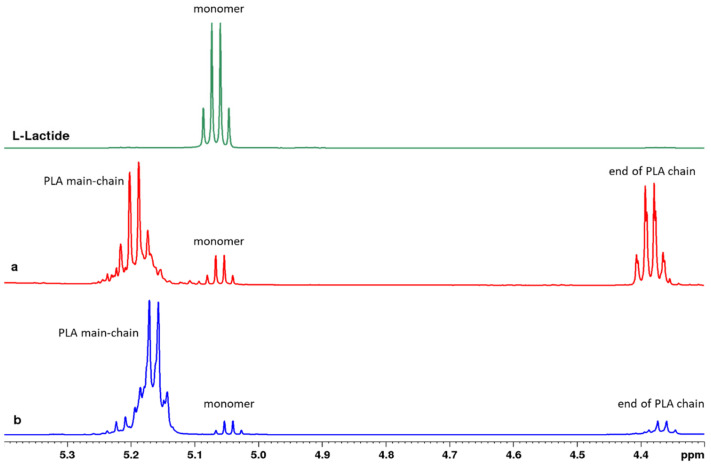
^1^H-NMR spectra of L-lactide and the reaction product from bulk enzymatic ROP with 0% CRL (**a**) and 5% CRL (**b**) at 100 °C, 72 h.

**Figure 3:**
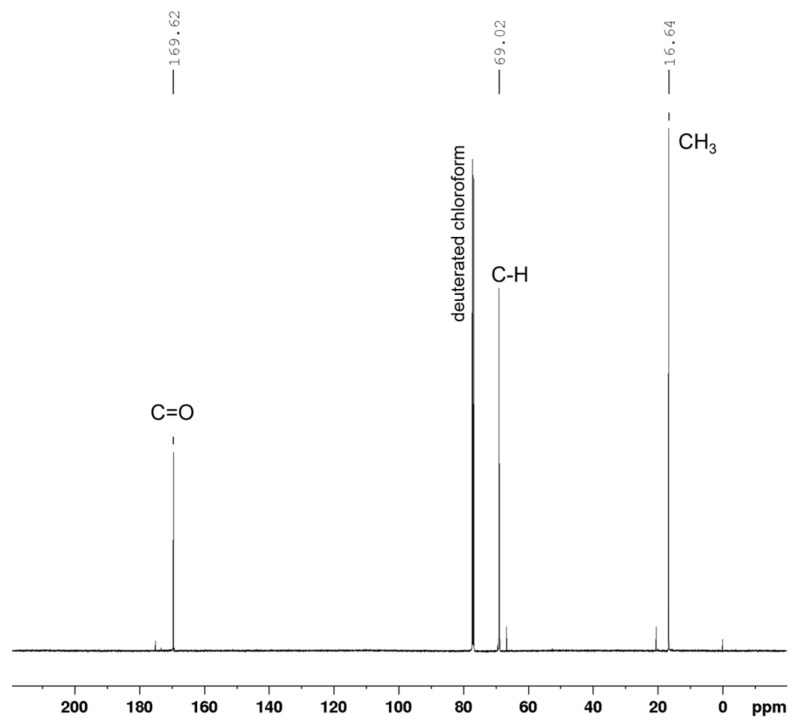
^13^C-NMR spectra of the reaction product (PLA) obtained from bulk eROP with 5% CRL at 100 °C, 72 h. Marked peaks are the signals coming from the PLA main chain.

^1^H-NMR spectra of the sample reacted without CRL (Figure 2a) indicated that some degree of polymerization could arise by heating the reaction system to 100 °C. However, the product showed a relatively strong signal at a chemical shift of 4.36–4.41 ppm, a quartet splitting coming from the -CH- bond of the end chain. It indicated that without CRL, the products were short-chain oligomers, which was also confirmed by the GPC result (Table 1, Data 1). Conversely, although 100 °C is considered relatively high for enzymatic reaction, this study confirmed that the presence of CRL aided the formation of longer PLA chains. This is evidenced by the higher main-chain to end-chain ^1^H-NMR peak intensity ratio, as opposed to without CRL (Figure 2b vs. Figure 2a). The GPC result (Table 1) also supported this finding. The ^13^C-NMR spectra of the PLA obtained from bulk eROP with 5% CRL (Figure 3) also confirms the formation of the polymer. It clearly shows the characteristic peaks of the PLA main chain at 169.62 ppm (C=O), 69.02 ppm (C-H), and 16.64 ppm (CH_3_), which agrees very well with another study [29].

Increasing CRL concentration for the bulk eROP resulted in higher conversion and molecular weight. This phenomenon could also be observed visually from the change in viscosity of the reaction mixture. When no CRL was used, there was no visible change in viscosity after 72 h. On the other hand, with 5% CRL, the change in viscosity during the reaction was obvious. The melted lactide was liquid at the beginning of the reaction. It progressively turned more viscous and hardened on the third reaction day. Such solidification was also observed in other studies on the eROP of L-lactide, especially at low temperatures or in solvent-free systems [11,15,16].

The hardening is likely caused by rheological limitation at the temperature used due to the presence of certain amount of polymer within the reaction system. For bulk eROP, such limitation could be reduced by using higher polymerization temperature. However, this was not performed in this study to avoid severe enzyme denaturation. Nonetheless, such hardening also indicated that longer PLA chains were formed when 5% CRL was used than those with lower CRL concentrations, justifying the role of CRL in the polymerization process.

Since the achievable molecular weight is likely to be limited by the rheology of the system, increasing CRL concentration at the same temperature would be misleading. For the same reason, prolonging the reaction time to more than 72 h could not be studied for bulk eROP at 100 °C. Hence, in this study, 5% CRL concentration and 3 days of reaction at 100 °C were taken as the optimum operating condition for the bulk eROP using CRL. The optimum condition obtained would be used as the operating condition for most of the other experiments in this study. Although certain reactions could finish at different reaction times depending on the condition of the mixture, to observe the effect of one factor at a time (OFAT), a similar operating condition would be used for the rest of this study.

The molecular weight achieved from the CRL-catalyzed eROP here was still relatively low compared to most other studies (Table 1). However, this could be attributed to the shorter reaction time and lower reaction temperature used in this study. The optimum condition obtained here also differs from an earlier study on CRL-catalyzed eROP of crude lactide. They used a CRL concentration of 1–10% *w*/*w* at a temperature of 70–130 °C. The type of CRL used was the same as in this study. Their optimum operating condition was 2% CRL concentration at a temperature of 90 °C. They used crude lactide (unpurified lactide) synthesized in-house with a purity of 81% [30]. The remaining content is the prepolymer and possibly other compounds from the depolymerization process [31]. Using crude lactide may explain the possibility of performing the eROP at 90 °C in their study. Whereas for L-lactide with high purity, bulk ROP should only be possible above 95–98 °C.

It may also be interesting to note that for bulk eROP in this study, the reaction system and the CRL only showed slight color change during eROP. This could indicate that the relatively high reaction temperature, compared to typical enzymatic reactions, did not severely damage the CRL. Indeed, an earlier study suggested that the temperature for the thermal denaturation of enzymes increases with decreasing associated water. In other words, the enzyme would denature at a significantly higher temperature in an anhydrous environment versus in an aqueous solution [32]. They showed that CRL denatured at 118 °C in a system with a water activity (a_w_) of 0.33, while in water, its denaturation temperature was only 61 °C [32,33].

### 3.2. The Effect of Solvents

Using solvents in the enzymatic reaction can be beneficial as they can reduce the viscosity of the reacting mixture. This could allow the reaction to proceed at a lower temperature, providing a more thermal-friendly environment for the enzyme. An appropriate solvent could also help obtain a higher molecular weight than a solvent-free eROP [15,19]. However, the opposite situation could also occur.

#### 3.2.1. The Effect of Solvents’ Hydrophilicity

Most of the commonly used organic solvents in chemical synthesis are hydrophilic [18] and have a rather low boiling point (<100 °C). Hence, choosing appropriate solvents to study their effect on the eROP of L-lactide was difficult. In this study, the solvents used were DMA (log P −0.77 [22]), 1,2-dimethoxybenzene (log P 1.6 [34]), 1,4-dimethoxybenzene (log P 2.04 [35]), diphenyl ether (log P 4.21 [36]), dodecane (log P 6.1 [37]), and the ionic liquid [BMIM][PF_6_] (log P −1.03 [38]). They were chosen by considering their boiling point (above 150 °C), safety, and hydrophilicity (in the form of log P).

During the eROP, crystals also formed in the upper part of the reaction tubes in the systems whose solvent was diphenyl ether, dodecane, and [BMIM][PF_6_]. No crystal was formed for the rest of the solvents, i.e., DMA and the dimethoxybenzes. The formed crystals (see Section 3.1) were also dissolved using chloroform and mixed with the reaction product to obtain accurate monomer conversion. The mixing was carried out in the respective reaction tube. An additional 2.5—5 mL of chloroform was sufficient to completely dissolve the formed crystals.

**Figure 4:**
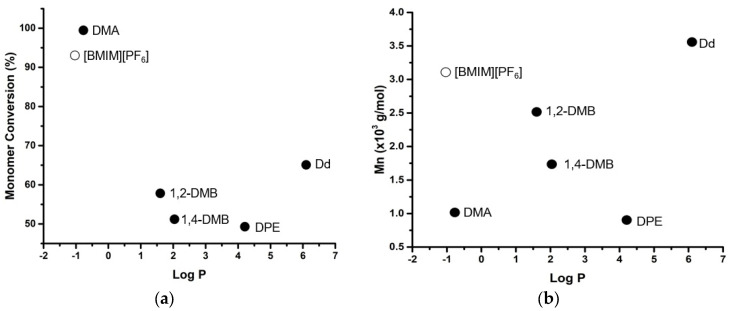
The effect of log P of the solvents (organic solvents (•) and [BMIM][PF_6_] (○)) on L-lactide conversion (**a**) and number average molecular weight (Mn) of the PLA (**b**) in the eROP of L-lactide catalyzed by 5% CRL (100 °C, 72 h, monomer to solvent ratio (g:mL) of 2:1). Abbreviations: 1,2-DMB—1,2-dimethoxybenzene; 1,4-DMB—1,4-dimethoxybenzene; DPE—diphenyl ether; Dd—Dodecane.

Our results (Figure 4a) show that L-lactide conversion does not follow the S-shaped behavior described by earlier studies [18,19,20]. The highest monomer conversion was achieved when low log P solvents were used. This is partly in line with an earlier study where DMA and [BMIM][PF_6_] also gave high monomer conversion [15]. 

**Figure 5:**
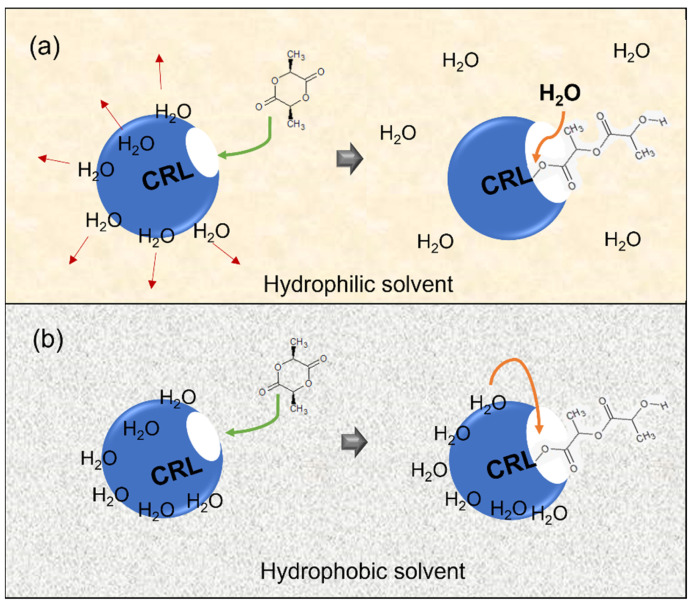
The influence of hydrophilic/low log P solvent (**a**) and hydrophobic/high log P solvent (**b**) on water molecules surrounding the CRL. Water molecules that become better distributed in the reaction system and high temperature prompt a faster initiation rate (lactide ring opening) in hydrophilic solvent (**a**) than in hydrophobic solvent (**b**).

The high conversion here might be because of the ability of the low log P solvents to pull the water molecules surrounding the enzymes, and together with agitation, the water molecules become more well distributed in the reaction system (Figure 5). Considering the proposed mechanism for eROP of lactide and lactones in earlier studies [39,40], such a phenomenon may lead to faster initiation due to the availability of water molecules as initiators near the enzyme-activated monomers. This, coupled with the higher reaction temperature used here (100 °C) compared to typical enzymatic reactions as well as in the eROP of ε-caprolactone in previous studies [19,20], might cause a fast enough initiation rate to consume most of the lactide monomers in [BMIM][PF_6_] and DMA-mediated eROP. However, in the DMA-mediated one, the molecular weight was low. In a previous study on bulk eROP of ε-caprolactone, the influence of water content on substrate conversion and polymer molecular weight has been elucidated. Higher water content leads to higher conversion. Nevertheless, both too high and too low water content resulted in low polymer molecular weight. The former is due to the possibility of hydrolysis, and the latter case is the consequence of enzyme rigidity [19].

The molecular weight of the obtained PLA here generally still follows the common trend. Hydrophilic solvents tend to induce lower enzymatic activity, thus lower molecular weight, and the opposite happened for hydrophobic solvents (Figure 4b). Exceptions occur on the eROP of L-lactide in [BMIM][PF_6_] and diphenyl ether. Indeed, the log P of diphenyl ether falls near the category where its effect on enzymatic activity is unpredictable (log P 2.0–4.0) [18]. The relatively high Mn of the obtained PLA in [BMIM][PF_6_]-mediated eROP catalyzed by CRL is discussed in the later part of this study (Section 3.4).

The log P of [BMIM][PF_6_] mentioned in this study is the log P at saturation [38]. In a common organic solvent, a log *p* value of −1.03 would normally mean that the solvent is hydrophilic. Other literatures, in fact, reported different (lower) log P values for [BMIM][PF_6_] [38,41]. However, those studies might not ponder ionic liquids log P’s dependence on its initial concentration [38]. In any case, compared to other ionic liquids, [BMIM][PF_6_] is considered hydrophobic [23,24]. Although the common measure of properties that have been well accepted for organic solvents is not completely suitable to accurately predict the behavior of ionic liquids [24], many times, enzymes tend to preserve their activity in hydrophobic ionic liquids compared to the hydrophilic ones [23].

At last, it is interesting to note that Figure 4 also indicates that solvents with log P between 2–4 seem to lead to low monomer conversion and molecular weight. Toluene, whose log P is 2.7, has been shown as a poor solvent for the eROP of L-lactide in earlier studies, in which no polymerization of L-lactide could take place or only a very low molecular weight of PLA was formed [9,14,21].

#### 3.2.2. The Effect of Monomer-to-Solvent Ratio

Even though the eROP of L-lactide in dodecane yielded the highest Mn of PLA, the L-lactide melt and dodecane were immiscible. Such immiscibility in solvents with high log P was also observed in other studies [19,20]. This made the reaction mixture behave more like bulk because the substrate and the product are insoluble in the solvent [19]. Due to the immiscibility of the reaction system mediated by dodecane, the effect of the monomer-to-solvent ratio was performed using [BMIM][PF_6_] as the reaction media.

The above study was done with a monomer-to-solvent ratio of 2:1 (g/mL). Considering that the bulk eROP of L-lactide by CRL (Section 3.1) resulted in higher molecular weight than eROP with solvents in the study above, the monomer-to-solvent ratio was investigated with decreasing solvent content. The amount of L-lactide in each reaction mixture was maintained the same.

**Figure 6:**
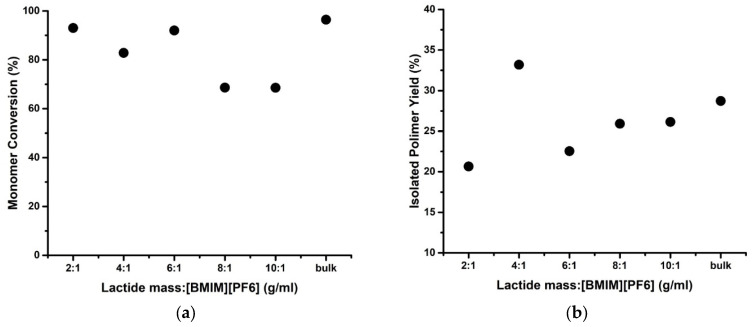
The effect of monomer-to-solvent ratio (g/mL) on L-lactide conversion (**a**) and the isolated PLA yield (**b**) in the eROP of 1 g of L-lactide catalyzed by 5% CRL at 100 °C, 72 h.

A decrease in the amount of [BMIM][PF_6_] leads to a decrease in L-lactide conversion, except in the solvent-free system (Figure 6a). A similar trend was observed in another study on the eROP of ε-CL in toluene using Novozym-435. The monomer conversion increased with increasing solvent content until a certain ε-CL concentration. This was presumably due to the better partitioning of the polymer product in the system with enough solvent. Thus, the polymer product is not accumulated in the enzyme’s active sites, giving better accessibility to the monomer and allowing a faster initiation rate in a system with higher solvent content [42]. However, this does not explain the higher conversion in the solvent-free eROP (‘bulk’ in Figure 6a). Comparing the bulk and the solvent-mediated eROP, enzymes in the bulk eROP would only collide with the monomer or the formed oligomer/polymer without passing through other solvent molecules. Therefore, the melted L-lactide is thought to be more accessible in solventless systems. The melted lactide might also provide sufficient partitioning for the oligomer/polymer formed in the bulk eROP so as not to saturate the active site of the enzymes, especially in the early stage of the reaction.

**Figure 7:**
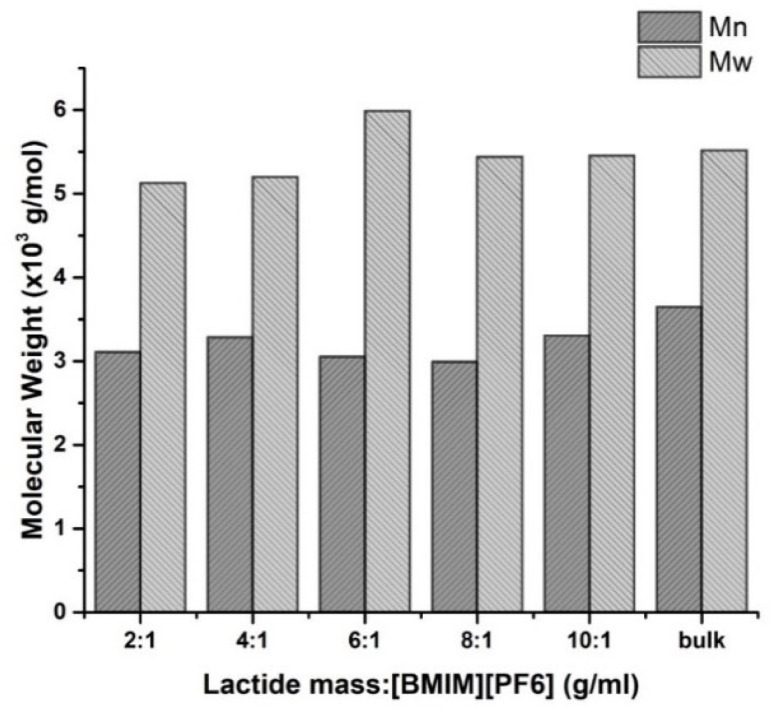
The molecular weight of the PLA with varying L-lactide-to-[BMIM][PF_6_] ratio (g/mL) in the eROP of 1 g of L-lactide catalyzed by 5% CRL at 100 °C, 72 h.

Unlike the trend in monomer conversion, the isolated polymer yield and the molecular weight were generally increased with lower [BMIM][PF_6_] content (Figure 6b and Figure 7). The smaller amount of solvent increases the probability for the enzyme-activated monomer/polymer chain to meet another opened chain. Thus, the chance of prolonging the polymer chain through the propagation reaction is also increased.

### 3.3. CRL-Catalyzed eROP in Bulk vs. in [BMIM][PF_6_]

At the same temperature, catalyst ratio, and reaction time, the CRL-catalyzed eROP of L-lactide in [BMIM][PF_6_] yielded PLA with similar molecular weight as that produced in bulk (Table 1, Data 3 vs. Table 2, Data 2). Compared to the case of varying [BMIM][PF_6_] content above, the propagation is thought to be easier in solvent-free systems due to the absence of solvent molecules. However, it is undoubted that the viscosity would be higher in bulk eROP.

As expected, the use of [BMIM][PF_6_] allows the eROP of L-lactide to be performed at a lower temperature compared to bulk ROP (Table 2, Data 1). However, only oligomeric chains were formed. No precipitation was observed when the product-chloroform mixture of this sample was precipitated in cold methanol, confirming the low molecular weight of the sample. Increasing the temperature to 100 °C significantly improved the monomer conversion and the molecular weight.

Compared to other studies of [BMIM][PF_6_]-mediated eROP (Table 2), the obtained molecular weight here falls in the medium range. This could potentially be improved if the temperature and reaction time are increased. As can be seen from Table 2, in a study using [HMIM][PF_6_], prolonging the reaction time from 3 to 7 days significantly increased the molecular weight of the PLA [11]. The highest Mn reported on ionic liquid-mediated eROP was 54,600 g/mol when [BMIM][BF_4_] was used as the solvent [8]. However, another study obtained a molecular weight of only 2700 g/mol with the same solvent, even at a higher eROP temperature [15]. Some other ionic liquids that have been used in the eROP of L-lactide are also shown in Table 2.

### 3.4. The CRL-Catalyzed eROP Mediated by [BMIM][PF_6_]

[BMIM][PF_6_] is one of the most commonly used ionic liquids in enzymatic reactions [24]. Compare to [BF_4_]^−^ anion, [PF_6_]^−^ is less nucleophilic [23]. Hence, [BMIM][PF_6_] would normally be a safer ionic liquid choice for CRL compared to the also commonly used [BMIM][BF_4_]. This is because enzymes would be more susceptible to denaturation when surrounded by stronger nucleophiles [23]. CRL exhibited good activity in [BMIM][PF_6_]-mediated reactions and even showed better performance, e.g., higher reaction rate, enantioselectivity, and or thermostability, versus in organic media [26,27,43,44,45].

The use of CRL in [BMIM][PF_6_]-mediated ROP resulted in better monomer conversion compared to ROP without CRL (Table 2, Data 2–3). Nonetheless, the molecular weight and the polydispersity were similar. The question then arose of whether or not the CRL catalyzed the eROP in this ionic liquid media. Before continuing, it is important to note that from the bulk eROP study (Section 3.1), it was clear that CRL could catalyze the eROP in solvent-free systems.

The potential cause of higher conversion in [BMIM][PF_6_]-mediated ROP with CRL has been described in Section 3.2.1. Moreover, extra water molecules were available from the enzyme for systems containing lipase, in contrast to systems without CRL. Enzymes typically have essential water surrounding their structure, allowing them to maintain activity in an anhydrous environment [46]. Data 2 and 3 from Table 2, as well as Data 1 from Table 1, likely indicate that [BMIM][PF_6_] could act as a catalyst in the ROP of L-lactide. [BMIM][PF_6_] probably took part in the polymerization because the imidazolium cations might induce cationic propagation [17]. However, in another study, no polymerization was observed when lipase was not added to the [BMIM][PF_6_]-mediated ROP of L-lactide [15]. Hence, it is unclear whether the catalytic effect was given solely by the [BMIM][PF_6_] or was also due to the CRL. Different perspectives were used and described in the following paragraphs to elaborate on this issue.

Unlike in bulk, a significant color alteration was observed in the presence of [BMIM][PF_6_]. In a sample without CRL (Table 2, Data 3), the transparent reaction mixture turned light brown by the end of the reaction. However, the crystals formed at the upper part of the tube were already dark brown within 24 h. As described earlier, crystals were formed in the upper part of the tube for the eROP in bulk and some of the solvents due to the reactor configuration used. The color change became more pronounced in CRL-catalyzed systems mediated by the ionic liquid. However, during the monomer-to-solvent ratio study, it was also noticed that the browning decreased significantly with decreasing [BMIM][PF_6_] content. Hence, the color alteration phenomena seem to be more caused by the [BMIM][PF_6_]. It is also important to note that when the CRL-catalyzed eROP in [BMIM][PF_6_] was conducted at 80 °C, no browning was observed, both in the reaction mixture and the formed crystals.

Thus, it is suspected that at 100 °C, the ionic liquid might undergo decomposition in this study. Apparently, upon contact with water, hexafluorophosphate anion could hydrolyze; and one of the decomposition products, HF, could denature enzymes [24,47]. Nonetheless, [BMIM][PF_6_] has been one of the most used ionic liquids in enzymatic reactions. A study on [PF_6_]^−^ decomposition inferred that [PF_6_]^−^ hydrolysis occurred particularly at low pH or elevated temperature. While at room temperature, the anion was relatively stable, even after prolonged storage. The study was conducted by contacting [BMIM][PF_6_] to the equivolume of water and leaving them to equilibrate [48]. To limit the amount of water, our system was pre-purged with Argon for 10 min before starting the reaction. However, the presence of water might be unavoidable in the eROP system that at 100°C, the [PF_6_]^−^ started to hydrolyze. Argon was chosen over nitrogen because Ar is denser than air. Thus, it is expected to remain in proximity to the reacting mixture’s surface throughout the reaction. The opened lactide ring during the initiation might also reduce the pH in the system, which could promote the hydrolysis of the [PF_6_]^−^. This could also partially explain the similar molecular weight in the [BMIM][PF6]^−^ mediated ROP with and without CRL (Table 2, Data 2 and 3), that the decomposition product might denature the CRL in the system.

We performed several simple tests to check the possibility of [BMIM][PF_6_] hydrolyzing or decomposing at 100 °C. 1 mL of [BMIM][PF_6_] was put in four closed test tubes along with 10% water, 20% water, 1 mL lactic acid, or a mixture of 1 mL lactic acid and 1 mL water. Each tube consists of a different mixture. These tubes were not pre-purged with Ar. They were placed in an oil bath at 100 °C for several days. In systems with 10% and 20% water, the condensed water was immediately visible on the inner wall of the upper tube, which is in contact with ambient air. No change was observable in these two ionic liquid systems even after 5 days. This could be because the added water was relatively little or because the water immediately evaporated when the system was submerged in the oil bath. No sufficient contact was made between the water and the [PF_6_]^−^ ions. In the other two systems, lactic acid was added to resemble the pH effect of the opened lactide ring as one of the triggers for [BMIM][PF_6_] hydrolysis. In systems containing lactic acid, the ionic liquid mixture became whitish, and a white layer appeared on the inner wall of the test tubes within a few hours. In the tube with 1:1:1 [BMIM][PF_6_]: lactic acid: H_2_O, crystals were also formed in the upper part of the tube within the first few hours. After 4 days, part of the white layer in 1:1 [BMIM][PF_6_]: lactic acid tube turned slightly brown. The whitish compound and the crystals formed in these tubes could not dissolve when mixed with chloroform. They had better dispersibility upon contact with acetone but still could not dissolve. The formed crystals also did not dissolve in water. These indicated that the formed compounds were not PLA, lactic acid, or lactide. All isomers of PLA (PLLA, PDLA, PDLLA) would normally dissolve in chloroform [49]. As such, it can be inferred that the formed compounds could be the decomposition products resulting from the contact of [BMIM][PF_6_] with lactic acid/opened lactide ring and water at 100 °C. The browning observed in our [BMIM][PF_6_]-mediated eROP, especially at 2:1 and 4:1 L-lactide to [BMIM][PF_6_] ratio was more pronounced compared to those done in the test tubes. This also suggested that the higher pressure resulting from the presence of Ar and the closed system in our eROP system might also contribute as the trigger for the browning/[PF_6_]^−^ decomposition in the [BMIM][PF_6_]-mediated eROP of L-lactide.

## 4. Conclusions

This study shows that *Candida rugosa* lipase (CRL) can catalyze high-purity L-lactide’s enzymatic ring-opening polymerization (eROP). Here, the effect of solvent hydrophilicity on the eROP of L-lactide was assessed in terms of monomer conversion and molecular weight of the PLA. The effect of solvent’s log P on L-lactide conversion did not follow the typical trend for enzymatic reaction, particularly because the water molecules are needed in the ROP mechanism, and the eROP of L-lactide could only be performed at elevated temperature (≥100 °C). Nevertheless, the obtained molecular weight of the PLAs still followed the common effect of log P on enzymatic transformations. Using ionic liquid [BMIM][PF_6_] as solvent for the eROP resulted in better monomer conversion and molecular weight than the organic solvents-mediated eROP studied here. However, by varying the amount of [BMIM][PF_6_], it was observed that the polymer yield and the molecular weight would improve when the [BMIM][PF_6_] content was decreased. Although [BMIM][PF_6_] allows the eROP to proceed at a lower temperature, a higher temperature was still needed to reach an acceptable molecular weight. This study also pointed out that hydrolysis of [BMIM][PF_6_] could occur at 100 °C even in the presence of a small amount of water, possibly because the opened lactide might lower the system’s pH and due to the high pressure of the closed system.

## Figures and Tables

**Table 1:** Bulk (solvent-free) enzymatic ring-opening polymerization (eROP) of L-lactide using *Candida rugosa* lipase (CRL) (this study) and comparison with other studies.

Data	T [°C]	% Enzyme ^a^	Time [days]	Conv. [%] ^b^	Mw [g/mol]	Mn [g/mol]	PDI	Yield [%]	Ref.
1	100	0	3	43.4	330	229	1.44	n.a. (l) ^c^	this study
2	100	2	3	55.4	4771	3332	1.43	n.a. ^d^	this study
3	100	5	3	59.3	5519	3649	1.51	29	this study
Other studies ^e^:									
Enzyme									
LPS/BCL	100	10	7	-	-	3500	1.14	22	[13]
	100	3	7	82	48,000	-	1.2	8	[6]
	125	10	7	98	-	78,100	1.4	-	[12]
N435	92	10	7	100	-	8800	-	7.2	[11]
	100	6	10	-	2440	-	2.6	91	[7]
	130	20	7	72.2	16,900	-	1.75	14	[15]
CALB	130	10	-	-	-	40,000	1.13	54	[8]
CRL	90	2	3	-	5428	2854	1.9	93	[30]

^a^ Relative to the mass of L-lactide. ^b^ Monomer conversion was estimated from ^1^H-NMR spectra. ^c^ Product only available in liquid form (no precipitate was formed). ^d^ Yield was not measured. ^e^ Value with a “-“ sign in the comparison tables indicates that the value of this variable was not mentioned in the respective reference. Abbreviations: LPS—Amano Lipase PS; BCL—*Burkholderia cepacia* lipase; N435—Novozym 435; CALB—*Candida antarctica* lipase B; CRL—*Candida rugosa* lipase.

**Table 2:** Enzymatic ring-opening polymerization (eROP) of L-lactide in [BMIM][PF_6_] with a monomer-to-solvent ratio of 2:1 (g/mL), catalyzed by CRL (this study) and comparison with other studies.

Data	Enzyme	T [°C]	% Enzyme ^a^	Time [days]	Conv. [%] ^b^	Mw [g/mol]	Mn [g/mol]	PDI	Ref.
1	CRL	80	5	3	70.9	501	431	1.16	this study
2	CRL	100	5	3	93.2	5126	3108	1.65	this study
3	CRL	100	0	3	66.2	5441	3196	1.70	this study
Other studies ^c^:									
Ionic liquid									
[BMIM][PF_6_]	N435	65	10	11	-	-	581	1.2	[16]
	CALB	120	10	1	-	-	3900	1.19	[8]
	N435	130	20	7	93.5	17,000	-	1.71	[15]
[HMIM][PF_6_]	N435	90	10	3	-	-	2800	1.5	[11]
		90	10	7	-	-	37,800	1.3	[11]
[BMIM][BF_4_]	CALB	110	10	1	96.2	-	54,600	1.25	[8]
	N435	130	20	7	53.3	2700	-	1.37	[15]
[BMIM][Tf_2_N]	CALB	120	10	1	90.4	-	50,100	1.42	[8]
	N435	130	20	7	83.7	14,100	-	1.28	[15]
[CH_3_(OCH_2_CH_2_)_3_-et-Pip][OAc]	N435	130	20	7	62.1	30,900	-	1.14	[15]
[Choline][Tf_2_N]	N435	130	20	7	60.9	1400	-	1.19	[15]

^a^ Relative to the mass of L-lactide. ^b^ Monomer conversion was estimated from ^1^H-NMR spectra. ^c^ Value with a “-“ sign indicates that the value of this variable was not mentioned in the respective reference. Abbreviations: [BMIM][PF_6_]—1-butyl-3-methylimidazolium hexafluorophosphate; [BMIM][BF_4_]—1-butyl-3-methylimidazolium tetrafluoroborate; [HMIM][PF_6_]—1-hexyl-3-methylimidazolium hexafluorophosphate; [BMIM][Tf_2_N]—1-butyl-3-methylimidazolium bis(trifluoromethylsulfonyl)imide; [CH_3_(OCH_2_CH_2_)_3_-et-Pip][OAc]—1-ethyl-3-(2-(2-methoxyethoxy)ethoxy)ethyl)piperidinium acetate; [Choline][Tf_2_N]—Choline bis(trifluoromethylsulfonyl)imide.

## Data Availability

The data presented in this study are available from the corresponding author upon request.

## Abbreviations

[BMIM][BF_4_]—1-butyl-3-methylimidazolium tetrafluoroborate; [BMIM][PF_6_]—1-butyl-3-methylimidazolium hexafluorophosphate; CRL—*Candida rugosa* lipase; DMA—N,N-dimethylacetamide; 1,2-DMB—1,2-dimethoxybenzene; 1,4-DMB—1,4-dimethoxybenzene; DPE—diphenyl ether; Dd—Dodecane; ε-CL—ε-caprolactone; eROP—enzymatic ring-opening polymerization; log P—logarithm of partition coefficient; Mn—number average molecular weight; Mw—weight average molecular weight; NMR—nuclear magnetic resonance; OFAT—one factor at a time; PLA—polylactic acid; PLLA—poly-L-lactic acid; THF—tetrahydrofuran.
